# How well do blood folate concentrations predict dietary folate intakes in a sample of Canadian lactating women exposed to high levels of folate? An observational study

**DOI:** 10.1186/1471-2393-7-25

**Published:** 2007-10-25

**Authors:** Lisa A Houghton, Kelly L Sherwood, Deborah L O'Connor

**Affiliations:** 1 School of Nutrition & Dietetics, Acadia University, 12 University Avenue, Wolfville, NS, B4P 2R6, Canada; 2 Department of Nutritional Sciences, University of Toronto, Toronto, ON, M5S 3E2, Canada; 3 Department of Clinical Dietetics and Physiology and Experimental Medicine Program, The Hospital For Sick Children, 555 University Avenue, Toronto, ON, M5G 1X8, Canada

## Abstract

**Background:**

In 1998, mandatory folic acid fortification of white flour and select cereal grain products was implemented in Canada with the intention to increase dietary folate intakes of reproducing women. Folic acid fortification has produced a dramatic increase in blood folate concentrations among reproductive age women, and a reduction in neural tube defect (NTD)-affected pregnancies. In response to improved blood folate concentrations, many health care professionals are asking whether a folic acid supplement is necessary for NTD prevention among women with high blood folate values, and how reliably high RBC folate concentrations predict folate intakes shown in randomized controlled trials to be protective against NTDs. The objective of this study was to determine how predictive blood folate concentrations and folate intakes are of each other in a sample of well-educated lactating Canadian women exposed to high levels of synthetic folate.

**Methods:**

The relationship between blood folate concentrations and dietary folate intakes, determined by weighed food records, were assessed in a sample of predominantly university-educated lactating women (32 ± 4 yr) at 4-(n = 53) and 16-wk postpartum (n = 55).

**Results:**

Median blood folate concentrations of all participants were well above plasma and RBC folate cut-off levels indicative of deficiency (6.7 and 317 nmol/L, respectively) and all, except for 2 subjects, were above the cut-off for NTD-risk reduction (>906 nmol/L). Only modest associations existed between total folate intakes and plasma (r = 0.46, *P *< 0.001) and RBC (r = 0.36, *P *< 0.01) folate concentrations at 16-wk postpartum. Plasma and RBC folate values at 16-wk postpartum correctly identified the quartile of folate intake of only 26 of 55 (47%) and 18 of 55 (33%) of subjects, respectively. The mean RBC folate concentration of women consuming 151–410 μg/d of synthetic folate (2^nd ^quartile of intake) did not differ from that of women consuming >410 μg/d (3^rd ^and 4^th ^quartile).

**Conclusion:**

Folate intakes, estimated by food composition tables, and blood folate concentrations are not predictive of each other in Canadian lactating women exposed to high levels of folate. Synthetic intakes > 151–410 μg/d in these women produced little additional benefit in terms of maximizing RBC content. More studies are needed to examine the relationship between blood folate concentration and NTD risk. Until data from such studies are available, women planning a pregnancy should continue to consume a daily folic acid supplement of 400 μg.

## Background

Folic acid supplementation during the periconceptional period is effective in reducing the risk of neural tube defects (NTDs) [1-5]. However, the proportion of Canadian women that report consuming a folic acid-containing supplement during the periconceptional period is low ranging between 17 – 28% [6-9]. Similar rates are documented even among women who are knowledgeable about the health benefits of consuming folic acid during this life-stage [8]. As a result, mandatory folic acid fortification of white flour and select cereal grain products was implemented in 1998 in Canada with the intention to increase the dietary folate intakes of reproducing women by 80 to 100 μg/d [10]. Indeed folic acid fortification has resulted in a dramatic increase in blood folate concentrations in North America, and a significant reduction in NTD-affected pregnancies (19 – 78%) [7,11-14].

In response to the improved blood folate concentrations of reproductive age women, many health care professionals are requesting direction on how aggressively they should promote folic acid supplementation among patients with high blood folate concentrations during the periconceptional period. The research literature shows many Canadian women post-fortification have red blood cell (RBC) folate concentrations approaching or exceeding 906 nmol/L [7,15], a value in early pregnancy shown to be an indicator of a reduced risk for NTDs [16-18]. For example, we previously reported median RBC folate concentrations ≥ 3000 nmol/L during the 3^rd ^trimester of pregnancy in a sample of well-educated Canadian women [15].

It must be acknowledged that the body of evidence supporting a relationship between folic acid supplementation during the periconceptional period and the risk of an NTD-affected pregnancy is considerably more robust than that of the association between blood folate concentrations and NTD risk [1-5,19]. Nonetheless, it is hard to argue against the logic that RBC folate concentrations, a measure of tissue folate stores, are unlikely to be elevated unless dietary folate intakes are similarly high [20]. For example, in controlled feeding studies preceded by a folate depletion phase of 2–7 weeks, serum and RBC folate concentrations of reproductive age women reflect the folate content of their diet (folate depletion, 400, 800 μg/day dietary folate equivalents [DFE]) [21-25]. Pre-folic acid fortification of the food supply, we reported a weak association between RBC folate concentrations and dietary folate intakes in young women not consuming a folic acid-containing supplement (r = 0.23, *P *< 0.05) [26]. This association improved when young women consuming supplemental folic acid were included in the analyses (r = 0.50, *P *< 0.01), suggesting that as the proportion of folate intake from synthetic folic acid increases, so might the predictive value of blood folate measures in determining folate intake. We speculated that folic acid added as a fortificant to food may likewise strengthen this association given its stability, and improved precision and accuracy of measurement, compared to endogenous folates which are normally trapped within cellular matrices, and need to be converted to a microbiologically assayable form. Second, inclusion of women known to consume supplements and fortified foods is likely to increase the range of exposure to folate which may also strengthen the association between blood folate concentration and folate intake. In contrast, if the relationship between blood folate concentration and folate intake is no longer linear at higher levels of exposure or the quality of the food composition tables has deteriorated given the variability in mandated versus actual levels of folic acid fortification, the association between blood folate concentration and dietary folate intake may not have improved.

Lactation is an opportune time to investigate the predictive value of blood folate concentrations in determining dietary folate intake as (1) women are clearly at a reproductive stage of their life cycle; (2) requirements for folate are elevated [27]; (3) vitamin supplement use is common. Therefore, the objective of this study was to examine whether, or not, blood folate concentrations, and dietary and synthetic folic acid intakes are predictive of each other in a sample of affluent, well-educated lactating women exposed to high levels of folate in the form of a supplement and fortificant.

## Methods

### Subjects

The dietary folate data and blood folate concentrations presented for the observational study presented herein were collected as part of a prospective, randomized control trial designed to assess the efficacy of supplemental [6S]-5-methyltetrahydrofolate ([6S]-5-methylTHF) versus folic acid during lactation (n = 69). A detailed description of the study design, procedures and information on the precision and reproducibility of laboratory procedures can be found elsewhere [15]. Briefly, participants were recruited between April 2002 and December 2003. Pregnant women (<36 wk gestation) were eligible for the overall study if they were healthy, non-smoking, between the ages of 16 to 40 years and intended to exclusively breastfeed for greater than 4 months postpartum. Subjects were identified by word-of-mouth and through the Motherisk Program, a telephone counseling service, at the Hospital for Sick Children, Toronto, Canada. The Human Ethics Committee of The Hospital for Sick Children approved the study and it was conducted in accordance with the policies and procedures of this institution and the Canadian Tri-Council policy statement on ethical conduct of research involving human subjects [28]. Participants gave written, informed consent.

### Study Design

At baseline (36 wk gestation), women were enrolled and a self-administered questionnaire was given to collect information on household income and education level. Participants were then given instructions on the completion of 3-d weighed food records and blood samples were collected. Three-d weighed food records and additional blood samples were collected at 4- and 16-wk postpartum (± 1 wk).

In the overall study, women were assigned to receive either a folate supplement (~400 μg/d of [6*S*]-5-methylTHF or folic acid) or a placebo within one week of delivery. Subjects were instructed to consume the study capsules daily until 16-wk postpartum and to avoid consuming any other folate-containing supplement during the intervention period. In addition all subjects received a daily folate-free multivitamin and mineral supplement, which contained 1 mg vitamin B6, 3 μg vitamin B12 and 4 mg ferrous fumarate (Exact; Pharmetics, Quebec, Canada).

### Laboratory procedures

Blood samples for the analysis of plasma and RBC folate were collected in EDTA-treated tubes at 36 wk gestation and at 4- and 16-wk postpartum. Plasma folate concentration is an indicator of recent dietary and supplemental folate intake whereas RBC folate concentration is reflective of tissue folate stores [20]. Hematocrit was measured immediately using freshly collected blood with the use of an electronic hematology analyzer (HmX, Beckman Coulter, Miami, Florida). Aliquots (100 μl) of whole blood were diluted 10-fold with ascorbic acid and ddH2O (1% wt:vol) and incubated at 37°C to allow for the deconjugation of folate. Plasma folate and RBC folate concentrations were measured by microbiological assay as described by Molloy and Scott [29]. Plasma homocysteine was measured by high performance liquid chromatography (HPLC) with electrochemical detection according the method of Cole et al. [30].

### Dietary Folate Assessment

Dietary folate intakes were estimated using 3-d weighed food records. Diet records were completed over 2 non-consecutive days and 1 weekend day by study participants who were trained by 1 of 2 registered dietitians on how to complete food records using an electronic digital scale accurate to 1 g (CS2000; Ohaus Corporation, Pine Brook, NJ). Dietary folate intakes were then tabulated from the diet records using Health Canada's Canadian Nutrient File (CNF), version 2001 b [31] which has been updated to reflect current mandated Canadian fortification levels and is based on the USDA Nutrient Database for Standard Reference [32]. The CNF reports dietary folate as (1) total dietary folate (μg) (sum of naturally occurring folate and folic acid added as fortificant) and (2) folic acid (μg), as a fortificant, separately. Supplemental folate intakes were determined by assessing the difference between the number of capsules dispensed at randomization (within 1 week postpartum) and number of capsules remaining at study completion (16-wk postpartum).

### Statistical Analysis

SAS for WINDOWS (version 9.1; SAS Institute Inc, Cary, NC) was used for statistical analyses and a probability level of 5% was chosen as the level of statistical significance. The large intra-subject variance in folate intakes were removed using SIDE: Software for Intake Distribution Estimation (version 1.0, Dept of Statistics and Center for Agricultural and Rural Development, Iowa State University, Iowa). The SIDE program operates within SAS for WINDOWS, and generates an estimate of subjects' usual (long-term average) intake by using the distribution of their observed intakes and partially removing the day-to-day variation in individuals' intakes.

Blood folate concentrations were normalized using logarithmic transformations. Partial correlations (*r*) were generated between plasma and RBC folate concentrations and folate intakes (total folate and synthetic folate, separately). In addition, the association between RBC folate concentrations at 16-wk postpartum and synthetic folate intake (by quartile assignment for each) was investigated using a one-way analysis of variance and Tukey multiple comparison procedures. RBC folate concentrations measured at study recruitment (36 wk gestation) and the form of folate supplement consumed (e.g. [6*S*]-5-MTHF, folic acid or placebo) during the intervention were entered as covariates in these latter analyses.

The effectiveness of using blood folate values to correctly classify folate intakes was assessed by comparing the quartile assignments for (1) dietary folate intake (total and synthetic folate) and (2) blood folate concentration (plasma and RBC folate) of each subject. When dietary and blood values were assigned to the same quartile, it was concluded that the blood folate value for that subject correctly classified their dietary folate intake. Dietary folate intakes misclassified by only one quartile were considered closely classified, and those misclassified by two or more quartiles were considered misclassified. If no association existed between the assigned quartile of blood folate concentration and dietary folate intake, one would expect 25% of participants to be correctly classified, 37.5% to be closely classified and 37.5% to be misclassified. The actual number of subjects assigned to each quartile was then compared with expected numbers using a chi-square goodness of fit test with 2 degrees of freedom.

Total dietary folate intakes are expressed in the manuscript, except where indicated, as "Dietary Folate Equivalents" [μg/d DFE = natural folate, μg + (1.7 × of supplemental folate, μg)] to adjust for the greater bioavailability of synthetic (added as a fortificant or supplemental) folate relative to endogenous sources [27]. For ease of interpretation, no adjustment was made to account for bioavailability of synthetic folate intakes when reported alone (e.g. expressed as μg/d, not μg/d DFE).

## Results

### Subject Characteristics

Only those subjects from the overall study who completed 3-d weighed food records and had provided a concomitant blood sample for folate analysis were included in the present analyses (4-wk postpartum, n = 53; 16 wks postpartum, n = 55). The mean age of the participants at enrollment was 32.2 ± 3.8 y (mean ± SD). Participants were well-educated (51 of 55 had completed college or university) and predominantly from households with incomes > $75,000 per annum. In Ontario, the mean household income in 2001 was estimated be $60,500 [33]. Prenatal supplements containing folic acid were used by 98% (54 of 55) participants during the course of their pregnancy. The average amount of folic acid provided by the supplements prenatally was 923 ± 239 μg/d.

### Biochemical indexes

Median blood folate concentrations during lactation were well above plasma and RBC folate cut-off levels indicative of deficiency (6.7 and 317 nmol/L, respectively) [34] (Table 1). In fact, even by 16-wk lactation, only two of the women had RBC folate values below 906 nmol/L, the value early in pregnancy shown to be an indicator of reduced risk of NTD. RBC folate concentrations of these two subjects were 869 nmol/L and 822 nmol/L at 16-wk postpartum. Neither woman consumed a folate supplement during lactation.

**Table 1:** Measures of folate status and estimation of folate intakes at 4 and 16 wk postpartum^1^

	Daily folate supplementation			
Variable	0 μg		400 μg	
	
	4 wks (n = 17)	16 wks (n = 18)	4 wks (n = 36)	16 wks (n = 37)
Plasma folate (nmol/L)	59 (41, 85)	46 (29, 63)	110 (74, 143)	93 (71, 120)
RBC folate (nmol/L)	2190 (1875, 2626)	1374 (1025, 1692)	3024 (2313, 3992)	2090 (1641, 2527)
% > 906 nmol/L^2^	100	89	100	100
Plasma homocysteine (μmol/L)	8.8 (8.1, 9.6)	8.7 (8.0, 9.4)	9.2 (8.6, 9.8)	8.9 (8.2, 9.6)
Dietary folate intake				
Synthetic folate, μg/d^3^	147 (75, 174)	118 (87, 151)	472 (408, 511)	450 (410, 479)
Dietary folate, μg/d	225 (193, 322)	253 (208, 320)	271 (214, 331)	268 (217, 326)
Total folate, μg DFE/d	481 (400, 561)	467 (379, 543)	1072 (983, 1163)	1040 (953, 1135)

Abbreviations: DFE, dietary folate equivalent = (μg of natural dietary folate) + (μg of folic acid from fortification × 1.7) + (μg of folate from supplement × 1.7)

^1 ^Median (1^st^, 3^rd ^quartile); ^2 ^RBC folate > 906 nmol/L is associated with low NTD risk **[16]**. ^3 ^Synthetic folate = supplemental + folic acid as fortificant

Moderate statistically significant correlations (r = 0.46–0.49, *P *< 0.001) were found between total (μg DFE) and synthetic folate intakes (μg) and plasma folate concentrations at 4- and 16-wk postpartum (Table 2). Weak, but statistically significant correlations (r = 0.31–0.36), were found between total (μg DFE) and synthetic folate intakes (μg) at 4- and 16-wk postpartum and RBC folate concentrations. Regardless of whether, or not, comparisons of folate intake were made with plasma or RBC folate concentration, the strength of the association between synthetic folic acid intake and blood folate values did not differ from the association when all sources of folate intake were considered in the analyses (i.e. naturally occurring + synthetic).

**Table 2:** Correlations between folate intakes estimated from a 3-day weighed food record vs. blood folate indexes

	4 wks			16 wks		
	
	n	r	*P*-value	n	r	*P*-value
**Total folate^1^**						
Plasma folate	53	0.47	< 0.001	55	0.46	< 0.001
RBC folate	53	0.32	< 0.05	55	0.36	<0.01
						
**Synthetic folate^2^**						
Plasma folate	53	0.47	< 0.001	55	0.49	< 0.0001
RBC folate	53	0.31	< 0.05	55	0.36	< 0.01

^1 ^Total folate = (μg of natural dietary folate) + (μg of folic acid from fortification × 1.7) + (μg of folate from supplement × 1.7)

^2 ^Synthetic folate = (μg of folate from supplement) + (μg of folic acid from fortification)

At 16-wk postpartum, plasma and RBC folate concentrations enabled correct prediction of the folate intake quartile assignment for only 47 and 33% of subjects, respectively (Table 3). Further, the percentage of participants correctly classified into their quartile of folate intake by plasma and RBC folate concentration diminished with the removal of supplement users.

**Table 3:** Percentage of participants correctly, closely or misclassified into quartiles of folate intake compared with classification by blood folate indexes

Variable	n	Correctly Classified	Closely Classified^1^	Misclassified^2^	P value^3^
**4 wks postpartum**					
Supplement users + nonusers					
Plasma folate	53	28	44	28	0.1427
RBC folate	53	40	40	20	< 0.001
Unsupplemented					
Plasma folate	17	18	41	41	0.2707
RBC folate	17	35	30	35	0.0588
**16 wks postpartum**					
Supplement users + nonusers					
Plasma folate	55	47	33	20	<0.0001
RBC folate	55	33	43	24	< 0.05
Unsupplemented					
Plasma folate	18	6	50	44	<0.0001
RBC folate	18	22	39	39	0.7866

^1 ^Percentage of participants misclassified by one quartile

^2 ^Percentage of participants misclassified by two or more quartiles

^3 ^A *P*-value < 0.05 indicates a rejection of the null hypothesis that there is no association between folate intakes and blood folate concentration based on the expectation that 25% of participants would be correctly classified, 37.5% closely classified and 37.5% misclassified. The actual numbers are compared with expected numbers using chi-square goodness of fit with 2 df.

**Figure 1:**
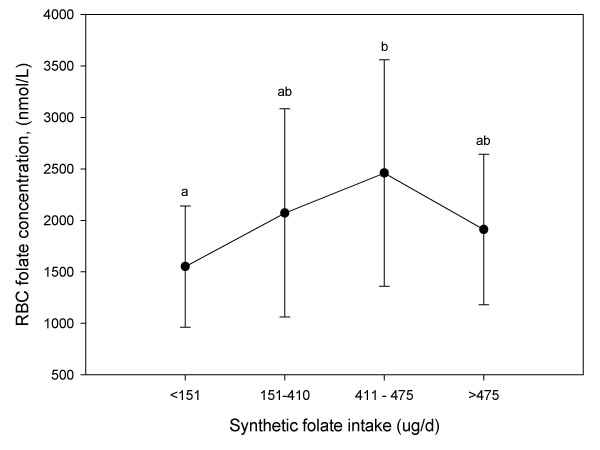
Mean RBC folate concentrations (± SD) by quartile of synthetic folate intake (n = 55) at 16 wk postpartum. Comparisons were made using one-way analysis of variance controlled for baseline RBC folate concentration (36 weeks gestation) and form of supplement provided during lactation (placebo, [6S]-5-methyl-THF, folic acid), and Tukeys multiple comparison procedures.

Mean RBC folate concentrations were plotted by the quartile of synthetic folic acid intake among lactating women16-wk postpartum to ascertain the mean RBC folate concentration consistent with supplemental folate intakes at or above 411 ug/d – the top two quartiles of synthetic folic acid intake (Figure 1). Given that women with synthetic folate intakes between 151–410 ug/d had mean RBC folate concentrations comparable to those in the upper two quartiles of folate intake suggests that the synthetic folate intakes >151–410 ug/d exceed their capacity to incorporate folate into erythropoiesis.

## Discussion

Results from the present study suggest that blood folate concentrations, and folate intake determined by food composition tables, are not reliable predictors of each other in a sample of Canadian lactating women exposed to high levels of folate. In fact, plasma and RBC folate concentrations predicted the correct quartile of folate intake of lactating women in our study less than half of the time. Arguably the best evidence supporting a relationship between folate and NTD-prevention comes from randomized prevention trials in which women were assigned to receive supplemental folic acid (± other vitamins and minerals) during the periconceptional period compared to those that were not [1,3-5]. Given the uncertainty of the mechanism(s) of how folate reduces the risk of NTDs, many health care professionals ask how well RBC folate concentrations predict synthetic folic acid intake in order to inform their decision on whether to recommend folic acid supplementation to women with very high blood folate concentrations. While it is conceivable that post-folic acid fortification of the food supply RBC folate concentration may be a better predictor of NTD risk than estimated folate intake, and that folic supplementation is no longer necessary for women with high RBC folate concentrations, randomized controlled trials to confirming this are unlikely to be conducted due to ethical considerations. Other experimental approaches will need to be considered. These known uncertainties together with our data illustrating that blood folate concentrations are not very predictive of folate intake suggest it would be prudent for women even with high blood folate concentrations to consume a folic acid supplement during the periconceptional period for NTD prevention.

Contrary to our original hypothesis, the strength of the association between dietary folate intake, determined by food composition tables, and blood folate concentrations has not improved post-folic acid fortification of the food supply. In the present study, statistically significant correlations between dietary folate intake, as determined by 3-d weighed food records, and RBC folate, an indicator of NTD risk, at 4- (r = 0.32, *P *< 0.05) and 16-wk (r = 0.36, *P *< 0.01) postpartum were found. The strength of these associations are very similar to those recently reported between dietary folate intake, as assessed by an abbreviated folate-targeted food/supplement screening tool, and RBC folate concentration, in a sample of women from California (r = 0.32, *P *= 0.0001) [35]. Neither the correlations reported in the present study nor those in the California report are stronger than those we previously reported pre-fortification in a group of young women from Southern Ontario (r = 50, *P *< 0.01) [26].

There are at least two likely explanations for our observations. First, the relationship between folate intake and blood folate concentration was no longer linear among lactating women in our study who for the most part had been chronically exposed to high levels of synthetic folate (Figure 1). Second, the quality of the food composition tables for folate is poor and, in fact their reflection of the "actual" folate concentration in foods may have deteriorated given the variability in mandated versus the actual levels of folic acid fortification. The actual folate content of the food supply in Canada is unknown. In 1998 when Health Canada mandated folic acid fortification of the food supply, they incorporated into the legislation an allowance for overages. In the U.S. where the food supply is also fortified with folic acid, overages are estimated to range from one to two times mandated levels [36-38]. In addition, the endogenous folate content of foods as listed in the version of the CNF used in our dietary analyses was determined prior to the use of the contemporary trienzyme folate extraction procedure; hence the quality of the folate values as listed in this database remains questionable and are very likely an underestimate of the true content of many foods. However, Han et al. too recently reported weak correlations between dietary folate intake and serum (r = 0.27) and RBC folate (r = 0.29) concentrations among healthy South Korean women despite the fact that they directly measured the folate content of most folate-containing foods after trienzyme extraction [39].

Regardless of the poor predictive ability of blood folate measures in assessing relative dietary folate intake in our study, it is clear that RBC folate concentrations in this group of affluent, well-educated lactating women are generally consistent with a reduction in risk of NTD (> 906 nmol/L) as proposed by Daly and colleagues [16]. Using data from a large case-control study of 56,049 women, Daly et al reported a greater than eightfold difference in NTD risk between women with RBC folate concentrations less than 340 nmol/L compared with those with levels of 906 nmol/L or higher (*P *< 0.001) [16]. In fact, only two women in the present study had RBC folate concentrations = 906 nmol/L at 16-wk postpartum; interestingly both were not consuming a folate-containing vitamin supplement during lactation. The RBC folate concentrations of these two women were 869 and 822 nmol/L, which is well above cut-off values for classic folate deficiency (363 nmol/L) [20,34].

As reported elsewhere, however, approximately one-third of this sample of lactating women had dietary folate intakes (folate endogenous to food + folic acid as a fortificant) below their estimated average requirement [40]. Assuming that folic acid is being added to the food supply at mandated levels, the synthetic folate intakes of women in the present study increased by 147 and 118 μg/d folate at 4- and 16-wk postpartum, respectively, as a result of folic acid fortification of the food supply. Together with the blood folate values reported herein, these observations suggest that as lactation continues beyond 16-wks, a greater proportion of women not consuming a folate supplement may develop RBC folate concentrations = 906 nmol/L. Subsequent to initiation of this study, Health Canada[41]., like the American Academy of Pediatrics [42] extended their recommended length of exclusive breastfeeding from 4–6 to 6 months (26 weeks). Thereafter, they recommend that infants receive complementary foods with continued breastfeeding through the first 12 months of life. In the present study, no women consuming a 400 μg/d folate supplement had a blood folate value ≤ 906 nmol/L or a folate intake below her estimated average requirement [40]. As illustrated in Figure 1, in this sample of women, there didn't appear to be an advantage of synthetic folate intakes > 151–410 μg/d in terms of maximizing RBC folate concentration. Considered together, then, these data suggest among well-nourished lactating women a folate supplement of 400 μg consumed consistently on a daily basis maximizes RBC folate content, and strikes the right balance versus higher levels (eg. 1000 or 5000 μg/d) of meeting the estimate average folate requirement of lactating women and not providing overly excessive amounts of folate than can not be incorporated into RBC precursors.

While these data are among the first to specifically assess how predictive blood folate concentrations and folate intakes are of each other post-folic acid fortification of the food supply, in addition to the quality of food composition tables, there are other limitations in estimating folate intake that must be acknowledged. Specifically, many women, particularly those that are overweight or concerned about their body weight, may under-report their dietary intake [43-45]. In addition, weighed food records, while generally regarded as the gold standard for determining dietary intakes may result in under-reporting of nutrient intake due to subject burden. Under-reporting and variability in measuring dietary intakes could have significantly weakened the statistical associations between folate intakes and blood folate concentrations in the present study. Both of the aforementioned issues could be of particular concern postpartum when many women have heightened awareness of their body weight and are busy with a new baby.

Finally, it should be noted that most lactating women in this study, on the advice of their primary care physician, consumed a 1000 μg/d supplement of folic acid during pregnancy. Many used the same folate-containing supplement prior to conception for NTD-prevention. While the life span of a RBC is ~120 days and hence by 16-wk lactation should reflect dietary and supplemental folate intakes during lactation, the contribution of liver folate stores amassed prior to parturition in meeting the nutritional requirements beyond 16 weeks of lactation could be significant. The absence of an improvement in the association between blood folate concentrations and folate intake between 4 and 16 weeks lactation could reflect this phenomenon. While the incremental increase in liver folate stores secondary to ingestion of 1000 μg/d of supplemental folic acid during pregnancy is not known, visceral folate stores in the form of long-lived pteroylpolyglutamate pools can be converted to pteroylmonoglutamates which can then be released into circulation [46,47].

## Conclusion

In this sample of affluent well-nourished lactating Canadian women chronically exposed to high levels of folate, plasma and RBC folate concentrations were poor predictors of relative folate intake, estimated by food composition tables generally, and synthetic folic acid intake specifically. Synthetic intakes > 151–410 μg/d in these women produced little additional benefit in terms of maximizing RBC content. More studies are needed to examine the relationship between blood folate concentration and NTD risk. Until data from such studies are available, women planning a pregnancy should continue to consume a daily folic acid supplement of 400 μg.

## Competing interests

The author(s) declare that they have no competing interests.

## Authors' contributions

DLO and LAH were responsible for the design of the study and wrote the manuscript. LAH and KLS recruited the subjects and were responsible for the sample collection, laboratory analysis and statistical analysis. All authors read and approved the final manuscript.

## Pre-publication history

The pre-publication history for this paper can be accessed here:


